# Genetic discrimination: introducing the Asian perspective to the debate

**DOI:** 10.1038/s41525-021-00218-4

**Published:** 2021-07-01

**Authors:** Hannah Kim, Calvin W. L. Ho, Chih-Hsing Ho, P. S. Athira, Kazuto Kato, Leonardo De Castro, Hui Kang, Richard Huxtable, Hub Zwart, Jonathan Ives, Ilhak Lee, Yann Joly, So Yoon Kim

**Affiliations:** 1 Asian Institute for Bioethics and Health Law, Yonsei University College of Medicine, Seoul, South Korea; 2 University of Hong Kong, Hong Kong, China; 3 Academia Sinica, Taipei City, Taiwan; 4 National University of Advanced Legal Studies, Kochi, India; 5 Department of Biomedical Ethics and Public Policy, Graduate School of Medicine, Osaka University, Osaka, Japan; 6 University of the Philippines, Quezon City, Philippines; 7 BGI-Shenzhen, Shenzhen, China; 8 Centre for Ethics in Medicine, University of Bristol, Bristol, UK; 9 Erasmus University Rotterdam, Rotterdam, The Netherlands; 10 Centre of Genomics and Policy, McGill University, Montreal, QC Canada

**Keywords:** Medical ethics, Health policy

## Abstract

Our article aims to provide a comprehensive portrayal of how seven Asian jurisdictions have sought to address the challenge of genetic discrimination (GD) by presenting an analysis of the relevant legislation, policies, and practices. Based on our findings, policy discussion and action on preventing or mitigating GD have been narrowly framed in terms of employment, insurance, disability, marriage, and family planning. Except for South Korea, none of the jurisdictions we examined has adopted specific legislation to prevent GD. However, for Asia to truly benefit from its recent scientific and technological progress in genomics, we highlight the need for these jurisdictions to engage more proactively with the challenges of GD through a coordinated regulatory and governance mechanism.

## Introduction

Genomic technologies have generated a wealth of new knowledge regarding health and disease. A wide spectrum of genetic tests—once only a figment of a distant reality—is becoming widely available, from whole-genome or -exome sequencing to multi-omics and epigenetic testing. These tests are being progressively integrated within healthcare systems, taken up in clinical practices, and offered as direct-to-consumer genetic testing (DTC-GT) services worldwide.

Asia in particular has taken its place at the forefront of the genomic and precision medicine revolution. Asian countries are active in large international collaborative projects such as the International Cancer Genome Consortium, the Global Alliance for Genomics and Health, and the International Human Epigenome Consortium. They have actively set up public biobanks with the big data extracted from their populations. For example, the Korean Genome and Epidemiology Study included ~245,000 participants’ samples with omics data in 2017^1^, and BioBank Japan gathered human DNA and serum samples from 291,274 participants in 2017^2^. In Taiwan, a National Biobank Consortium was established in 2020 to integrate existing 33 biobanks for a large and comprehensive biomedical big data network^3^ and the Hong Kong government has in 2020 established the Hong Kong Genome Institute to conduct large-scale genome sequencing^4^. Asia also has a vibrant private sector, which markets technologies and genetic services to consumers both within the region and across the globe.

While genetic testing may improve disease prediction, diagnosis, and treatment, the rapid uptake and application of genetics and genomics raise numerous ethical, legal, and social issues (ELSI). One of the most prominent among these is the growing number of possibilities of using genetic information to justify treating individuals differently or profiling specific population groups that may lead to genetic discrimination (GD). GD usually refers to the unfair and differential treatment of an individual based on actual or suspected genetic characteristics, and is recognized to arise in a number of contexts such as insurance, employment, and health care^5–7^. The recent processing of biological materials and genetics data biobanks and data repositories, along with the exponential growth of DTC-GT, across national borders, have signaled the need to re-initiate discussion of the ELSI on a global scale^8^.

By the first decade of the twenty-first century, several transatlantic studies documented GD in clinical settings^9^, life insurance^7,10,11^, and employment^12^. More recent studies vary considerably, ranging from those that present comparative data on the use of genetic testing results by private life insurance companies^13^ to the systematic reviews of international public policies and studies concerning GD^7^, and analyses of the perceptions and concerns regarding GD^14^. For instance, a public survey was conducted across Europe to examine apprehensions surrounding GD in the case of population-based genetic databases^15^. Another study examined public perception of GD associated with genetic testing in primary care, finding that perceptions substantially varied by nationality among US and Canadian residents^16^.

In contrast, there are only a few published studies on GD in Asia in which comparative law and policy approaches have been applied. Yoshizawa et al. discussed how genetic technologies might be used in discriminatory ways in six Asian jurisdictions, documenting a case of pre-employment GD in China^17^. Another study regarding genomic technologies and consent practices in Japan, Malaysia, and Taiwan highlighted the need to inform genomic research participants about the risk of GD^18^. In the light of growing collaborations in genomic research and the use of gene technologies across the Asian regions and beyond, our paper seeks to reinvigorate the use of comparative law and policy approaches to identify existing and emerging forms of GD and to harmonize legal and regulatory responses in ways that facilitate international collaboration.

## Materials and methods

This article begins with a review of legislation, policies, and practices related to GD in seven Asian jurisdictions—China (and Hong Kong Special Administrative Region (SAR)), India, Japan, Philippines, South Korea, Singapore, and Taiwan. These jurisdictions were selected primarily due to the legal and bioethical expertise available in the Asian Bioethics Network on Genomics, which was formed in 2016 to promote ethical dialog and regulatory harmonization across jurisdictions in Asia that have committed substantial resources to advance gene technologies and genomic medicine. All the contributing authors from these jurisdictions are members of the Network and have been involved in active and continuing dialog through in-person meeting in Seoul on April 16 and 17, 2018 and further electronic communication. These have in turn given rise to a degree of consensus on identifying and discussing GD across these jurisdictions that are highly varied in demographic composition, socio-political constitution, and level of technological development. The authors of the seven Asian jurisdictions conducted an in-country analysis of GD through policy and regulatory review using Google and official legal databases in the jurisdictions between January to May 2019. Further data searches were subsequently conducted by Y.J. and H.Kim during the period from May to July 2020.

This review is presented in two stages. In the first stage, an overview of the regulatory governance of GD in each jurisdiction is presented, particularly in relation to insurance, where concerns over prejudicial exclusion are widely recognized. The second stage is focused on outlining existing privacy frameworks that protect people against GD in these jurisdictions. Subsequently, this article highlights the progress of GD policies and the identified GD policy gaps to provide interpretations specific to the Asian context, before providing further comparisons with their European and North American counterparts. An emerging area of concern that we decided not to include in our manuscript is that of GD caused by the forensic use of genetic information and databases in Asia^19^. Because this type of use of genetic information does not create GD per se, but rather exacerbate existing types of discrimination based on other factors such as population group, citizenship status, wealth level, etc., it warrants a distinct investigation in future research.

### GD-directed legal, ethical, and political frameworks

The brief comparative review presented in this part illustrates the emerging legal and political trends developed to address GD in the selected Asian jurisdictions (Table 1). India and Mainland China address some occurrences of GD in courts of law, while others delegate the responsibility to national bodies to develop guidance on GD. Currently, only South Korea has enacted legislation that includes specific and broad provisions to prevent GD, the *Bioethics and Safety Act* (BSA). In addition, measures protecting people against GD have been included in research oversight frameworks, such as national ethics guidelines and research funding policies.

**Table 1:** Laws and policies that apply to GD and protect genetic privacy.

Jurisdiction	Laws and policies regarding GD	Laws and policies that protect genetic privacy
China	Measures for the Administration of Health Insurance (2019)	Personal Information Security Specification (2020)—genetic information as sensitive information Regulation of the People’s Republic of China on the Administration of Human Genetic Resources (2019)
Hong Kong SAR	Disability Discrimination Ordinance (1996), as applied by the court in K & Others v Secretary for Justice, (unreported) [2000] DCE03, 4, 7/99	Personal Data Privacy Ordinance (1996)
India	M/S United India Insurance Co. v. Jai Parkash Tayal (2018) Ethical Guidelines for Biomedical Research on Human Subjects (2017)—for recommendatory	No (Personal Data Protection Bill (2019) has not yet been legislated)
Japan	Guidelines for collection and use of genetic information in health and life insurance companies (in preparation)	Act on the Protection of Personal Information (2017)—genetic information as personal information (personal identification code)
Philippines	National Ethical Guidelines for Health and Health-Related Research (2017)—warning GD as the potential harm to research participants Senate Bill 1875 (2013) (Act Prohibiting discrimination because of genetic information in health insurance coverage) has not legislated	Data Privacy Act (2012)—genetic data as sensitive personal information
South Korea	Bioethics and Safety Act—prohibits GD in education, employment, promotion, insurance, or any other social activity	Personal Information Protection Act of 2011 (2017)—genetic information as sensitive information
Singapore	National guidelines on Genetic Testing and Genetic Research by the Bioethics Advisory Committee (2005)—recommendation against unfair discrimination particularly in insurance	Genetic Testing and Genetic Research (2005)—genetic data in medical practice and research Personal Data Protection Act (2012)—genetic data as personal information if it identifies an individual or it combined with other personal information Code of Practice issued by the Ministry of Health has regulatory effect in 2020
Taiwan	The Constitution (Art 7)—regarding the equality right in general Employment Service Act (1992)—prohibits employment discrimination based on disability	Personal Data Protection Act (2012)—genetic data as sensitive personal data

#### China (and Hong Kong)

The China Banking and Insurance Regulatory Commission (CBIRC) adopted new government guidelines—the Measures for the Administration of Health Insurance (MAHI)—in 2019, to prohibit GD in health insurance^20^. Under the Insurance Act (2015), the CBIRC mandates that business licenses be issued to both state-owned or private insurers, including foreign-funded insurers^20^. The MAHI applies to those types of insurance companies and their employees^20^. Specifically, the MAHI prohibits insurance companies from “mak[ing] differentiated pricing” of an insured person based on genetic information and genetic testing results (art. 17). The MAHI also prohibits insurance companies from collecting and requesting genetic testing results of an insured person “when an insurance company sells health insurance products” (art. 38). However, the MAHI allows the use of the “family genetic medical history” of the insured person for purposes of differentiated pricing of the insured person or collecting genetic information when selling health insurance products (arts. 17 and 38).

In Hong Kong SAR, the *Disability Discrimination Ordinance* and the *Personal Data (Privacy) Ordinance* provide some protection against GD, although neither statutes are specific to genetics or gene technology. Under this statute, a court has ruled that it is unlawful for the civil service to discriminate against potential or actual employees based only on a family history of mental illness related to genetic testing results^21^. In a recently published blueprint to drive the local development of genomic medicine, an expert group appointed by the SAR government indicated that more specific regulatory measures may be needed to prevent GD in life, critical illness, and health insurance^22^. Considerable emphasis has also been placed on public engagement in the blueprint, which may support better identification of GD and means to address related concerns.

#### India

A 2018 High Court of Delhi case—*M/S United India Insurance Co. v. Jai Parkash Tayal*—highlights the pervasive effect of GD as it relates to private health insurance and employment^23^. The Court concluded that such discrimination did not adhere to proper standards of genetic testing and failed to comply with the test of *intelligible differentia*, a standard of natural justice and equity (The Constitution of India). However, the Supreme Court of India stayed this judgment and prevented the operative part of the Delhi High Court’s judgment from transforming India’s laws regarding GD^24^. Indeed, the majority of employers and insurance providers in the country are in the private sector, and impleading the insurance regulator, the Insurance Regulatory Development Authority of India, is the right step to address potential GD in the insurance sector.

The Indian Council of Medical Research developed the *National Ethical Guidelines for Biomedical and Health Research Involving Human Participants* in 2017^25^. The guidelines affirm that social stigmatization and discrimination in education, employment, health care, and general insurance are probable consequences of the irresponsible use and sharing of genetic research. The guidelines also specify the need to obtain informed consent for secondary uses of genetic information and prescribe norms relating to the maintenance of data confidentiality, as well as pre- and post-test counseling. However, these guidelines do not legally bind the scientific community, serving instead as ethical guidelines.

#### Japan

Japan does not have a law regulating discrimination based on personal genetic traits. While the *Fundamental Principles of Research on the Human Genome* issued in 2000 by the Bioethics Committee of the Council for Science and Technology addresses this topic, its normative strength is limited. The Principles state that “…regardless of their genetic characteristics, all individuals or groups of individuals are respected for their dignity and human rights, and that they are equal to one another and are not subjected to any form of discrimination.”^26^ The subsequently adopted government guidelines for human genome research—*Ethical Guidelines for Human Genome/Gene Analysis Research* (2001)—do not mention GD^27^.

However, recently, more active initiatives have emerged in Japan. In 2019, the Japanese insurance industry announced that guidelines banning life insurance companies from collecting or using personal genetic information for purposes related to coverage eligibility or premium calculations are being planned to prevent GD^28,29^. In 2019, a non-partisan group of lawmakers approved the draft outline of a Bill to promote responsible use of patients’ genetic information in disease treatment and to prevent GD^30^.

#### The Philippines

The latest unsuccessful attempt to directly address GD in health insurance was the Senate *Bill 1875* in 2013, entitled *An Act Prohibiting Discrimination because of Genetic Information in Health Insurance Coverage*^31^. The Bill was meant to prevent insurers from discriminating based on predictive genetic data in the context of both individual and group health insurance contracts. The *National Ethical Guidelines for Health and Health-Related Research* (2017) expressed concerns that “there is potential harm to research participants arising from the use of genetic information, such as stigmatization or discrimination.” This is why “researchers shall take special care to protect the privacy of participants and confidentiality of such information.”^32^

#### Singapore

The Ministry of Health issued the *Code of Practice* for the provision of clinical and laboratory genetic services that have regulatory force from 2020 onwards^33^. In the Code, all licensed clinical laboratories providing laboratory genetic/genomic testing services are prohibited from disclosing genetic test results without consent to any third parties, including family members, although some limited exceptions may apply. However, insurance companies usually require such consent to be provided in application forms to purchase personal insurance (e.g., life, health, and disability). In biomedical research (such as the SG10K pilot study), the use of genetic technologies and information is mainly governed under the *Personal Data Protection Act* (PDPA) and *Human Biomedical Research Act*, which implements recommendations of the Bioethics Advisory Committee^34^.

#### South Korea

Under the *Constitution of the Republic of Korea*, the *National Human Rights Commission of Korea Act* proscribes discriminatory acts violating equality rights, including those based on disability or medical history (art. 23)^35^. However, genetic features are not explicitly included under the terms “disability” and “medical history” in the provision.

However, the BSA (2004), the first legislation to explicitly prohibit GD in Asia, seems to alleviate the above concerns. The Act stipulates that discrimination “on the grounds of genetic information in education, employment, promotion, insurance, or any other social activity” (art. 46-1) is illegal, and prohibits “compelling any person to undergo a genetic test or to submit the results of a genetic test” (art. 46-2)^36^. Furthermore, the BSA contains a criminal sanction for those who discriminate based on genetic information with imprisonment up to 2 years or fines of up to 30 million KRW (art. 67-1-4)^36^.

#### Taiwan

GD, to some extent, is linked with the recognition of the indigenous rights of the aboriginal groups in Taiwan. Under the *Human Subjects Research Act*^37^ and the *Basic Law of Indigenous Peoples*^38^, *Regulations of Consulting and Obtaining the Consent and Participation of Indigenous Peoples and Tribes* have recently been implemented to avoid ethnically related stigmatization of Taiwanese aboriginal groups in the context of genetics studies and biomedical research^39^.

Otherwise, GD in Taiwan could be proscribed according to a broad interpretation of the equality right stipulated in the *Constitution* (art. 7) (The Constitution of Taiwan, 1947). However, when encountering certain cases, it is not appropriate to apply the *Constitution* directly without invoking a specific law. Protection against GD in the workplace may be available through a similarly broad interpretation of the *Employment Services Act*, which prohibits discrimination in employment based on several characteristics such as race, religion, and disability (art. 5)^40^. Nevertheless, GD is not explicitly addressed under the Act, and there are yet to be judicial decisions regarding GD under this legislative scheme.

### Genetic privacy governance against discriminatory practices

Among the general privacy legislation, the *Enforcement Decree of the Personal Information Protection Act* (PIPA) in South Korea, the PDPA in Taiwan, and the *Data Privacy Act* (DPA) of 2012 in the Philippines stipulate genetic data as sensitive personal data (Enforcement Decree of the PIPA art. 18(1), PDPA art. 2(1), DPA Sec. 3(t))^41–43^. In addition, in the reformed 2017 *Act on the Protection of Personal Information* in Japan, genetic information is interpreted as a personal identification code similar to sensitive information^44^. These are similar to the *General Data Protection Regulation* in the European Union that specifies special categories of personal data, including genetic data^45^. Although personal genetic data that can cause an infringement of the rights of the subject through data leakage is categorized as sensitive information or personal identification code in those laws, the laws require additional safeguards to process sensitive data. For instance, the laws provide specific security and safeguard measures for personal data controllers who process such sensitive personal data. They make it more difficult for personal genetic data to be used in a way that renders them vulnerable to stigmatization and discrimination, but these safeguards do not directly refer to GD. As the other example, there also has been no case to test the illicit use of genetic or health information presented to the Personal Information Protection Commission, which performs privacy impact assessment under the PIPA in South Korea^46^.

The *Personal Information Security Specification* (PISS)—the major digital privacy governmental guidelines in China—also protects individual genetic information, to some extent^47^. If personal biometric and health information can be a source of personal reputational damage or discrimination, it is categorized as personal sensitive information (PISS art. 3(2)). Compared to the data protection laws in South Korea and Taiwan that explicitly specify genetic data as sensitive personal data, PISS perceives genetic information as personal biometric and health information. PISS also includes more exceptions to confidentiality besides authorized consent, such as national security and national defense, public safety, public health, a criminal investigation, prosecution, trial, and judgment enforcement (PISS art. 5(4)).

Another type of genetic privacy governance can be seen in Japan and Singapore. In the PDPA in Singapore, genetic information is perceived as personal information only if it identifies an individual on its own or when combined with other information^48^. This is similarly the case in Hong Kong SAR under the Personal Data (Privacy) Ordinance^49^.

In the limited contexts of clinical or research testing, including biobanking, some legislation and regulations provide rules to protect the confidential nature of genetic information; however, those laws are also silent on the topic of GD. For instance, China’s *Regulation of the People’s Republic of China on the Administration of Human Genetic Resources* in 2019 provides for the respect of the privacy rights of human genetic resources providers in collecting, preserving, utilizing, and providing the resources (art. 9)^50^. Further, it prohibits the collection of human genetic resources of genetically related families without ethical approval issued by the national authorities (art. 11). If anyone violates the provision of the Regulation, the authorities and the Ministry of Science and Technology of China can order that the illegal act be stopped and illegally collected human genetic resources be confiscated, with the rule breakers paying a fine of up to five million yuan (art. 36).

In South Korea, the *Medical Service Act of 1962* (2019) provides a rule that prevents any disclosure or transmission of a patient’s data to a third party without the patient’s consent (art. 21-2(1))^51^. However, this does not apply to genetic data held outside clinical records^51^. The BSA protects the confidentiality of genetic information in medical records, preventing disclosure to anyone other than the patient (art. 46-3)^36^.

The biobanks in Taiwan, which hold genetic data and samples from Taiwanese citizens, benefit from a governance structure set by the *Human Biobank Management Act* enacted in 2010^52^. The Act establishes a privacy protection framework for the storage of, use of, and access to genetic samples and data contained therein. However, this legislative effort has not extended its protection to GD.

## Discussion

The results of our review showcase the existing GD policy framework in selected Asian jurisdictions, raising important questions about their comprehensiveness and effectiveness. South Korea enforces the legislative prohibition of GD with criminal sanctions, but these have never been tested in a court of law. Case law in India invalidated a discriminatory clause about genetic disorders in health insurance, but the persisting ambiguity of parameters of deniability in insurance plans, adequate regulation of the use, and sharing of health data, including genetic information, remains challenging. The recent government guidelines in China prohibit some discriminatory practices involving the collection and use of genetic information only in the context of health insurance. Other surveyed jurisdictions have chosen instead to address the GD topic in nonbinding ethical guidelines in research (India, Singapore, and Japan). Taiwan has broadly formulated the right to equality in its *Constitution*, which may afford its citizens some protection against GD, but this interpretation of the right to equality in relation to GD has not yet been tested in a court of law. Based on our findings, the protection against GD provided by policies and laws in the selected Asian jurisdictions varies significantly, from rather weak, partial, and uncertain, to relatively high (only in South Korea).

This review also tested whether existing policies and laws focusing on data protection provides some protection against GD. Surveyed jurisdictions show different coverage of genetic privacy to some extent (Table 1). The genetic privacy approach paralleled a growing interest in protecting the privacy of personal information in research. Our review found that legislative initiatives on protecting personal genetic data vary among the jurisdictions as the data are categorized into general personal and sensitive personal data depending on the context.

However, these legal and regulatory data privacy laws have not succeeded, so far, in providing a sufficiently strong degree of genetic nondiscrimination protection to individuals who use genetic testing services in Asia. These frameworks usually include standard exemption clauses, such as “with the consent of the individual to whom the information belongs,” that limit the extent of protection they provide. Specifically, no surveyed jurisdiction explicitly delimited the lawful use of genetic information or alleviated public concerns surrounding compelled, unexpected, or unrecognized disclosures of genetic data that can result in discrimination.

The lack of laws or policies against discriminatory use of genetic information may raise substantial concerns beyond clinical or research contexts. Discrimination in the workplace owing to genetic traits would be one area of concern. Although the International Labour Organization already argued for the protection of people with a genetic predisposition to developing diseases in the workplace back in 2007^53^, South Korea is the only Asian jurisdiction where GD in employment is punishable through a criminal sanction^36^. Specifically, there has been a reported case in pre-employment, *Xie v. Human Resources and Social Security Bureau in Foshan City*, adjudicated in mainland China. In 2009, 31 applicants to the Foshan local government were denied employment for being thalassemia gene carriers and three of them filed a lawsuit alleging discrimination^54^. However, in 2010, the Foshan Intermediate People’s Court ruled that it is legal to deny applicants who carry the thalassemia gene jobs in civil service^55^. Until now, there have been no guidelines regulating thalassemia genetic testing in pre-employment physical examination in the civil service as well as in state-owned companies. This situation has created some apprehension among job applicants, especially in southern China, where a high prevalence rate of α and β thalassemia carriers is reported (11%)^56^.

Compared to this, both the *Genetic Information Nondiscrimination Act* (2008)^57^ in the US and the *Genetic Nondiscrimination Act* (2017)^58^ in Canada currently prohibit the acquisition and use of genetic information for employment with criminal sanctions. Amendments to the Canadian Labour Code and the Human Right Code appoint the filing of GD claims to designated governmental agencies^59^. In the United Kingdom, the Information Commissioner’s Office, which oversees the *Data Protection Act*, has issued the *Employment Practices Code*^60^. This code prohibits the use of genetic testing information to predict a worker’s health condition, along with requests for disclosure of a worker’s genetic test results (4.5.1 and 4.5.2.)^60^. The code allows for exceptions where the genetic condition of a worker is likely to pose a serious safety risk to other people, or where a specific working environment or practice might pose risks to workers who have certain genetic traits (4.5.3)^60^.

In contrast to North America, concerns regarding GD in health insurance appear to be connected with universal health coverage in Asia. Japan, the Philippines, Hong Kong SAR, Singapore, South Korea, and Taiwan have adopted national health insurance systems in the form of compulsory social insurance since the latter half of the twentieth century. The systems have recently expanded their coverage range to reduce the financial burden of genetically at-risk individuals. In Taiwan, the tests subsidized by the national health insurance system are mainly for clinical diagnosis rather than early detection, which means only those with pre-existing symptoms of a genetic disease can receive a free diagnosis^61^. The *Selective Benefit Coverage System* under the *National Health Insurance Act* in South Korea has also covered the gene panel testing based on the next-genome sequencing for genetic disorders and cancers from 2019^62^. Since 2019, Japan has covered 70–90% of fees of the newly introduced cancer gene panel tests for intractable cancer patients, who have not responded to surgery and anticancer drug treatment^63^. PhilHealth—the National Health Insurance Corporation of the Philippines—extended its coverage of neonatal screening from six disorders to 28 additional disorders in the expanded program, and the confirmatory tests have included genetic testing since 2018^64^.

However, there remains limited evidence to identify the effectiveness of the public health coverage system against GD. From a 2019 national survey of public perception and knowledge of genomic research and medicine in Japan, 22% of respondents considered that the avoidance of discrimination in employment or insurance is important when personal genetic information is used in research and hospitals^65^. In particular, anxiety over GD in health insurance was considered low in the study, while GD in employment was a matter of concern for young participants.

Furthermore, little is discussed of the need to develop national policies and agreements to prohibit GD in the life insurance or private health insurance in Asia. A 2013 survey of 99 Korean physicians in genomics provided some evidence that the legal protection against GD for people with genetic risks was insufficient in the private health insurance at that time (50.5%)^66^. In Singapore, as the possibility of being denied insurance coverage is a common concern with genetic testing, the Life Insurance Association of Singapore declared in 2006 that its members would not require applicants to undergo genetic testing to obtain insurance coverage^67^. However, an important caveat to this policy statement is that individuals who are already aware of genetic test results relevant to their health must disclose this information to their insurer. There is currently no regulatory control over private insurers’ decision to extend insurance coverage, or not, to genetically at-risk individuals in the country. The recent move by the Japanese insurance industry to adopt GD guidelines is laudable, but independent monitoring and review of their implementation would provide added credibility to this initiative. A 2020 survey on the attitudes of insurance employees toward adverse selection and GD shows that it is necessary to improve understanding of GD through communication and consultation to prepare the guidelines for Japanese insurers^68^. As an example of a successful model, a 2008 agreement between the Association of Insurers, patient associations, and professional associations in the Netherlands led to the adoption of guidelines on mortality and morbidity risk determination owing to familial hypercholesterolemia. A 2012 tracking study confirmed that the guidelines facilitated access to life insurance, helped to reduce the negative perception about GD, and had been appropriately applied by insurers^69^.

Disability discrimination related to genetic traits remains a great concern in some Asian jurisdictions. When associated with the negative social perception identified in the Philippines and India toward patients with disabilities and rare diseases, such findings could reflect a trend toward stigmatization and marginalization of carriers of genetic diseases, which could be interpreted as GD^70–74^. A 1998 experts’ survey in China also showed that 64% of Chinese geneticists were aware of the lack of legal prohibition on discrimination based on disability^75^. Compared to this, Canada shows how the use of evidence of GD in a specific context, for example, that of Huntington’s disease^76^, as well as dynamic advocacy from patient organizations, can spark constructive debates surrounding the necessity of implementing legislation to prevent GD. In Canada, such debates led to the creation of the Canadian Coalition for Genetic Fairness and the adoption of Act S-201 (GNDA) to prevent GD, even outside the context of Huntington’s disease. Information presented in this context—in addition to the Huntington studies—included privacy surveys^77,78^, economic modeling of anti-selection risks in the event of a prohibition on the use of genetic results^79^, and research related to the life insurance industry practices^80^.

In addition, this review found some noticeable differences between the perception of GD in Asia and that in North America and Europe. In Asia, serious concerns exist regarding the possible use of genetic information when considering potential spouses for marriage and starting a family. In the 2017 Japanese public survey, the respondents were largely concerned about suffering potential disadvantage in the context of marriage and pregnancy (41.0%), employment (37.6%), and insurance (43.8%) owing to their genetic characteristics^81^. In a 2012 public survey of 1000 participants, 71.9% expressed that they would refuse to marry someone with genetic factors rendering them “high risk” for developing cancer^82^. In the aforementioned 1998 Chinese experts’ survey, a majority of Chinese geneticists agreed on the following: partners should know each other’s genetic status before marriage (92%); carriers of the same defective gene should not marry one another (91%); women should have prenatal diagnosis if medically indicated (91%); and governments should require premarital carrier tests (86%)^75^. Conversely, GD does not appear to be a significant concern when considering marriage or starting a family in North America and Europe, outside of specific population groups that are genetically at risk of developing a serious illness^83–85^.

This GD review is limited by several factors. First, our comparative assessment used only two approaches—the GD-specific frameworks and genetic privacy—so might not adequately identify more general concerns about GD in this region. A more broadly framed assessment would enable researchers and policymakers to understand and recognize how their nations provide some degree of protection from GD to their citizens with or without having to specifically regulate GD. Second, this review cannot be used to support or refute the view that incidents and concerns about GD are substantial in Asia. The reports on GD that we reviewed generally did not have a robust methodology or conveyed little evidence. This situation is not unique to Asia, as documenting GD has also been a struggle globally^86^. We encourage researchers to further document GD concerns and incidents in Asia using consistent, recognized, quantitative, and qualitative research methodologies. Third, we limited our comments on laws, policies, and practices by focusing on seven Asian jurisdictions. GD seems to have become a concern that merits policymakers’ attention in these jurisdictions, but this phenomenon could develop differently in other Asian regions.

## Conclusion

Our article aims to provide a comprehensive portrayal of how seven Asian jurisdictions have sought to tackle the challenge of GD. It provides an overview of laws, regulations, and policies adopted in these jurisdictions to address this complex issue. Unlike North America and Europe, where the GD debate was in full force by the late 1990s, in Asia, GD has generated fewer discussions to date reflecting both similar and distinct concerns from those observed in the western hemisphere.

We found that GD has called forth a variety of legal and political responses in Asia. However, this topic requires further investigation in order for countries in this region to be able to develop the kind of multilevel, nuanced, anti-GD policies required to account for different social, ethical, and economic factors. More specifically, the jurisdictions we reviewed have not yet succeeded in specifying the lawful use of genetic information or alleviating public concerns surrounding compelled, unexpected, or unrecognized disclosures of genetic data that can lead to discrimination.

For Asia to truly benefit from its innovative surge in genomics and precision medicine, such progress needs to be matched by a corresponding investment in addressing ELSI raised by genomics, predominant among which is GD. Our transnational collaboration could be the start of a promising regional discussion to ensure that issues are sufficiently presented and addressed in Asia. This would in turn drive the debate forward at the national level, thus promoting greater solidarity and fostering much-needed trust in science in this region.
